# Programmed death-1 promotes contused skeletal muscle regeneration by regulating Treg cells and macrophages

**DOI:** 10.1038/s41374-021-00542-4

**Published:** 2021-03-05

**Authors:** Jian Shou, Xinjuan Shi, Xiaoguang Liu, Yingjie Chen, Peijie Chen, Weihua Xiao

**Affiliations:** School of Kinesiology, Shanghai University of Sport, Shanghai, China

**Keywords:** Cell death and immune response, DNA adducts

## Abstract

Immune cells are involved in skeletal muscle regeneration. The mechanism by which Treg cells are involved in the regeneration of injured skeletal muscle is still unclear. The purpose of this study was to explore the role of programmed death-1 in contused skeletal muscle regeneration, and to clarify the regulation of programmed death-1 on Treg cell generation and macrophage polarization, in order to deepen our understanding of the relationship between the immune system and injured skeletal muscle regeneration. The results show that programmed death-1 knockdown reduced the number of Treg cells and impaired contused skeletal muscle regeneration compared with those of wild-type mice. The number of pro-inflammatory macrophages in the contused skeletal muscle of programmed death-1 knockout mice increased, and the expression of pro-inflammatory factors and oxidative stress factors increased, while the number of anti-inflammatory macrophages and the expression of anti-inflammatory factors, antioxidant stress factors, and muscle regeneration-related factors decreased. These results suggest that programmed death-1 can promote contused skeletal muscle regeneration by regulating Treg cell generation and macrophage polarization.

## Abstract (web summary)

Immune cells are involved in skeletal muscle regeneration, but the mechanisms are unclear. In this paper, the authors show that programmed death-1 (PD-1), a key immunosuppressive receptor that is involved in adaptive immune responses, can promote contused skeletal muscle regeneration by regulating Treg cell generation and macrophage polarization.

## Introduction

Skeletal muscle injury is a common injury in sports medicine [1], and skeletal muscle contusion is a common form of sports-related skeletal muscle injury [2]. Generally, the regeneration process in skeletal muscle injury is divided into three stages. The first stage occurs early in the injury, when the muscle fiber structure is destroyed, and there is swelling, necrosis, and a large number of infiltrating inflammatory cells. The second stage occurs 5–10 days after injury. At this time, muscle satellite cells are activated and proliferate, differentiate, and fuse with injured muscle fibers or residual myotubes to repair the damaged skeletal muscle. This stage is called the injury repair period. The third stage is the tissue shaping phase, which usually occurs 14–21 days after injury, in which regenerating muscle fibers mature [3].

Macrophages infiltrate into the injured site after skeletal muscle injury and play an important role in repairing injured skeletal muscle. It can specifically express macrophage antigen-2 (Mac-2) [4, 5]. There are two basic types of macrophages, pro-inflammatory and anti-inflammatory macrophages [6, 7]. In the early stage of skeletal muscle injury, pro-inflammatory macrophages mainly infiltrate into injured skeletal muscle [8]. Pro-inflammatory macrophages engulf necrotic muscle fibers and cell debris and secrete a variety of pro-inflammatory cytokines, such as tumor necrosis factor-α (TNF-α), interleukin-1β (IL-1β), interleukin-6 (IL-6), cluster of differentiation 86 (CD86), and inducible nitric oxide synthase (iNOS), which exacerbate an inflammatory response at the site of injury [9–13], CD86 and iNOS are pro-inflammatory macrophage markers [14, 15]. Subsequently, the number of anti-inflammatory macrophages increases, and anti-inflammatory macrophages secrete mechano growth factor (MGF), the anti-inflammatory factor interleukin-10 (IL-10), the profibrotic factor transforming growth factor-β (TGF-β), arginase 1 (Arg1), cluster of differentiation 163 (CD163), and mannose receptor (CD206) [10, 16–18], and they are also anti-inflammatory macrophage markers [14, 15], thereby promoting regeneration of the injured skeletal muscle [16]. Therefore, pro-inflammatory/anti-inflammatory polarization of macrophages plays an important role in repairing injured skeletal muscle.

Regulatory T (Treg) cells are a type of CD4^+^ CD25^+^ T lymphocyte that specifically express forkhead box P3 (Foxp3), and it is also Treg cells markers [19, 20]. Treg cells can be divided into two types, according to their source [21]: thymic Treg (tTreg) cells develop in the thymus through T cell receptor (TCR) interactions, which prevents autoimmunity, while peripheral Treg (pTreg) cells are produced by traditional peripheral CD4^+^ T cells [21], which limits their stimulus to microorganisms and nonmicrobial immune responses, maintaining immune tolerance [22] and inhibiting chronic allergic inflammation [21]. Studies have shown that pTreg cells gather in injured skeletal muscle and promote macrophage switching from pro-inflammatory to anti-inflammatory, thereby promoting the repair of injured skeletal muscle [19, 20, 23, 24]. Furthermore, pTreg cells also directly secrete the growth factor amphiregulin (AREG), thereby promoting muscle satellite cell proliferation [19, 20, 23, 25]. However, Treg cells accumulate in injured skeletal muscle, regulating macrophage polarization and affecting the regeneration of contused skeletal muscle, but the specific mechanisms have not been elucidated.

Other studies have shown that programmed death-1 (PD-1 or CD279) belongs to the CD28 superfamily and is a key immunosuppressive receptor that is involved in adaptive immune responses. PD-1 plays a key role in regulating autoimmunity, tumor immunity, virus/parasite immunity, the inflammatory response and allergies [26, 27]. Moreover, PD-1 inhibits the macrophage pro-inflammatory polarization and promotes the macrophage anti-inflammatory polarization [14]. Treg cells also express PD-1. PD-1 promotes the production of pTreg cells in peripheral tissues and has an important role in maintaining the normal function of pTreg cells [21, 28–30]. However, whether PD-1 is involved in contused skeletal muscle regeneration has not been elucidated. Based on this, we hypothesize that PD-1 can promote contused skeletal muscle regeneration by regulating Treg cell generation and macrophage polarization. Therefore, the purpose of this study is to explore the role of PD-1 in contused skeletal muscle regeneration, and to clarify the regulation of PD-1 on Treg cell generation and macrophage polarization, which will deepen our understanding of the relationship between the immune system and injured skeletal muscle regeneration.

## Materials and methods

### Animals

Forty normal male C57BL/6 mice at ≈8 weeks of age were purchased from Shanghai Southern Model Biological Research Center; 30 programmed death-1 knockout (PD-1 KO) male C57BL/6 mice (B6.Cg-Pdcd1tm1.1Shr/J) at ≈8 weeks of age were obtained from The Jackson Laboratory. The mice were housed at 21–25 °C, with 40–50% humidity, a 12-h light: 12-h dark cycle, and free access to drinking water and eating standard feed. Eight wild-type mice were randomly selected as the uninjured group (WT_con_), and the remaining mice were treated with gastrocnemius contusion and divided into the following groups: 1 day, 3 days, 7 days, and 14 days postinjury, with eight mice in each group. Of the PD-1 knockout mice, six were randomly selected as the knockout uninjured group (P_con_), and the remaining mice were treated with gastrocnemius contusion and divided into the following groups: 1 day, 3 days, 7 days, and 14 days postinjury, with six mice in each group. All the animals were not treated after gastrocnemius contusion. All animal experiments have complied with ARRIVE guidelines.

### Skeletal muscle contusion model and materials

After anesthetizing mice with 1.5% pentobarbital sodium (40 mg/kg), the knee joint was straightened by 0°, and the ankle joint was extended by 90°. A solid stainless steel ball with a mass of 16.8 g and a diameter of 15.9 mm was released at the top of a transparent tube (100 cm high, 16.0 mm inner diameter) and hit a striking device vertically, and the bottom of the striking device hit the middle part of the bilateral gastrocnemius muscle of the mouse (area: 28.26 mm [2]). After skeletal muscle contusion, gastrocnemius hematoma could be seen, and muscle fiber swelling, necrosis, erythrocyte exudation, and inflammatory cell infiltration were seen by HE staining. We previously verified this procedure many times, and this method has been successfully modeled [31–33].

After gastrocnemius contusion, samples were collected at different time points (1, 3, 7, and 14 days). The mice were sacrificed after anesthesia, and the bilaterally damaged gastrocnemius muscles were quickly collected. The control group samples were collected from the same position. In each group, paraffin specimens were prepared from the right gastrocnemius muscle for histomorphological examination, and the left gastrocnemius muscle was analyzed by fluorescent quantitative PCR.

### HE staining and Masson staining

After the gastrocnemius muscle of mouse was collected, it was fixed with 4% paraformaldehyde (China National Pharmaceutical Group Corporation), embedded in paraffin (China National Pharmaceutical Group Corporation), and then cut with a microtome (Leica‐EG 1160, Germany) to a thickness of 3–4 μm. After dewaxing, the sections were stained with hematoxylin–eosin (Google Biotechnology Co., Ltd.), and then mounted on neutral gum (China National Pharmaceutical Group Corporation) to obtain HE-stained sections. The sections were dewaxed and stained with Ponceau red (Google Biotechnology Co., Ltd.), and then mounted in neutral gum to obtain Masson-stained sections. The sections were observed and photographed under a 200× microscope, and the minimum diameter of regenerated muscle fibers in HE slices were analyzed by using ImageJ software.

### Immunofluorescence

The paraffin section was dewaxed, heated in a 95 °C water bath for 20 min in sodium citrate antigen-repair solution (Beyotime Biotechnology), and naturally cooled to room temperature. The sections were washed three times with TBS for 5 min each time and then washed with TBS-T for 5 min. The sections were blocked for 2 h at room temperature with blocking solution and incubated at 4 °C for 20 h with primary antibodies against Mac-2 (1:1000, Cedarlane, USA), iNOS (1:100, Abcam, UK), Arg1 (1:100, Cell Signaling Technology, USA), and Foxp3 (1:100, Cell Signaling Technology, USA). The sections were washed three times with TBS for 5 min each and then washed with TBS-T for 5 min. The sections were incubated with Alexa Fluor 555 (1:1000, Jackson, USA) and Alexa Fluor 488 (1:500, Jackson, USA) secondary antibodies at room temperature for 1 h in the dark. The sections were washed three times with TBS for 5 min each and then washed with TBS-T for 5 min. The sections were incubated with DAPI (Beyotime Biotechnology), washed three times for 5 min each with TBS and then washed with TBS-T for 5 min, and finally mounted with an anti-fluorescent quencher (Sangon Biotech (Shanghai) Co., Ltd.). The slides were observed and imaged with a confocal microscope (Zeiss LSM70) (depth is 4 μm). In total, 3–5 fields of view were taken per slice, the number of positive cells in each field of view was calculated and reported as a mean, for each independent experiment.

### Real-time PCR

The skeletal muscle was weighed (≈50 mg), cut, and placed in a 2 ml centrifuge tube, and 1 ml Trizol (Invitrogen) was added to extract RNA. cDNA synthesis was performed according to the instructions of the RevertAid™ First-Strand cDNA Synthesis Kit. A PCR instrument (Mastercycler EP, Eppendorf, Germany) was used for reverse transcription.

The real-time PCR system included 12.5 µl 2 × Maxima SYBR Green/ROX qPCR master mix (Vazyme, China), 1 µl cDNA, nuclease-free water, and 300 nM forward and reverse primers. The primers were synthesized by Sangon Biotech (Shanghai) Co., Ltd (Table 1). Amplification was performed using a real-time PCR instrument (ABI StepOnePlus Real-Time PCR System 7500, USA). The reaction conditions were as follows: predenaturation at 95 °C for 10 min, then 40 cycles of denaturation at 95 °C for 15 s, and 60 °C for 1 min. The ^△△^CT method was used to calculate the relative mRNA in the measured samples [34, 35].

**Table 1:** Primer sequences of quantitative RT-PCR.

PD-1	5′-GCCACCTTCACCTGCAGCTTGT-3′	5′-AAACCGGCCTTCTGGTTTGGGC-3′
Col1a1	5′-GAGCGGAGAGTACTGGATCG-3′	5′-GCTTCTTTTCCTTGGGGTTC-3′
Col3a1	5′-GTCCACGAGGTGACAAAGGT-3′	5′-GATGCCCACTTGTTCCATCT-3′
IL-1β	5′-CCCAAGCAATACCCAAAGAA-3′	5′-TTGTGAGGTGCTGATGTACCA-3′
TNF-α	5′-CTTCTGTCTACTGAACTTCGGG-3′	5′-CACTTGGTGGTTTGCTACGAC-3′
IL6	5′-GAACAACGATGATGCACTTGC-3′	5′-CTTCATGTACTCCAGGTAGCTATGGT-3′
IL4	5′-TACCAGGAGCCATATCCACGGATG-3′	5′-TGTGGTGTTCTTCGTTGCTGTGAG-3′
IL10	5′-GCTCTTACTGACTGGCATGAG-3′	5′-CGCAGCTCTAGGAGCATGTG-3′
TGF-β	5′- TGCGCTTGCAGAGATTAAAA-3′	5′- CGTCAAAAGACAGCCACTCA-3′
MCP-1	5′-GCTCAGCCAGATGCAGTTAAC-3ʹ	5′-CTCTCTCTTGAGCTTGGTGAC-3′
CXCL10	5′-CCAAGTGCTGCCGTCATTTTC-3′	5′-GGCTCGCAGGGATGATTTCAA-3′
Nox2	5′-TGAATGCCAGAGTCGGGATT-3′	5′-CGAGTCACGGCCACATACA-3′
Gpx4	5′-GCCTGGATAAGTACAGGGGTT-3′	5′-CATGCAGATCGACTAGCTGAG-3′
Prdx1	5′-CTGGCATGGATTAACACACCC-3′	5′-GGTGCGCTTGGGATCTGAT-3′
Sod1	5′-TATGGGGACAATACACAAGGCT-3′	5′-CGGGCCACCATGTTTCTTAGA-3′
IGF-1	5′-GCTTGCTCACCTTTACCAGC-3'	5′-AAATGTACTTCCTTCTGGGTCT-3′
MGF	5′-GCTTGCTCACCTTTACCAGC-3′	5′-AAATGTACTTCCTTTCCTTCTC-3ʹ
Pax7	5′-CTCAGTGAGTTCGATTAGCCG-3′	5′-AGACGGTTCCCTTTGTCGC-3ʹ
MyoD	5′-GAGCGCATCTCCACAGACAG-3′	5′-AAATCGCATTGGGGTTTGAG-3′
myogenin	5′-CCAGTACATTGAGCGCCTAC-3′	5′-ACCGAACTCCAGTGCATTGC-3′
Mac2	5′-CAGGACAGGCTCCTCCTAGTGC-3′	5′-CCAGCAGCAGGATAGCCTCCAG-3ʹ
iNOS	5′-CTGCAGCACTTGGATCAG-3′	5′-CGTACCAGGCCCAATGAG-3ʹ
CD86	5′-AGTGATCGCCAACTTCAGTGAACC-3′	5′-GGTGACCTTGCTTAGACGTGCAG-3′
Arg1	5′-GAACACGGCAGTGGCTTTAAC-3′	5′-TGCTTAGCTCTGTCTGCTTTGC-3′
CD206	5′-GGATTGTGGAGCAGATGGAAG -3ʹ	5′-CTTGAATGGAAATGCACAGAC -3′
CD163	5′-GCAAAAACTGGCAGTGGG-3′	5′-GTCAAAATCACAGACGGAGC-3′
AREG	5′-CCATGAAGACTCACAGCGAGGATG-3′	5′-ATGCCAATAGCTGCGAGGATGATG-3′
GAPDH	5′-ACTCCACTCACGGCAAATTC-3′	5′-TCTCCATGGTGGTGAAGACA-3′

### Statistical analysis

Two-factor repeated measurement analysis of variance was used to analyze statistical data, and *P* < 0.05 indicated significant differences. All data were analyzed using SPSS Statistics V21.0 software, and the results are expressed as the mean ± standard deviation.

## Results

### Increased PD-1 expression after skeletal muscle contusion

**Fig. 1:**
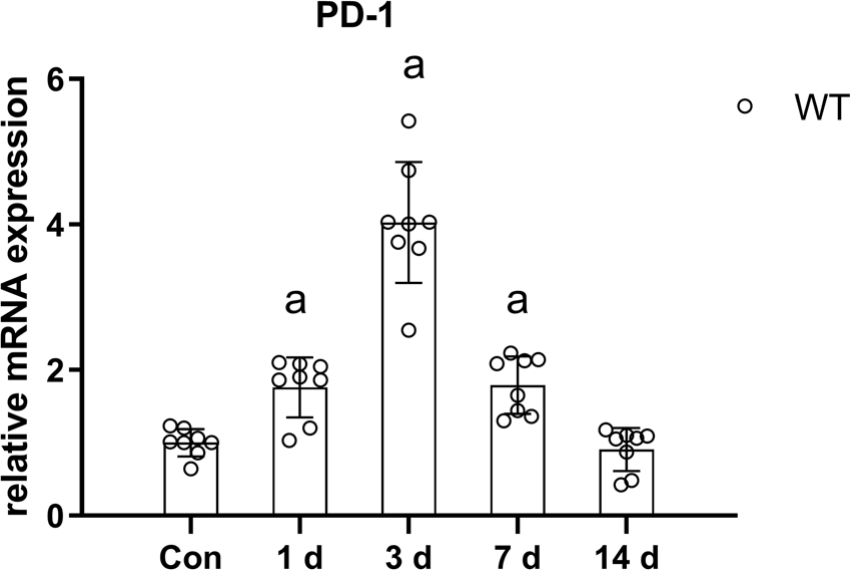
PD-1 mRNA expression in wild-type mice at various time points after skeletal muscle contusion. The data are mean ± SD, *n* = 8. ^a^ Compared with the WT_con_ group, *P* < 0.05.

The PCR results showed that PD-1 mRNA expression in wild-type mouse skeletal muscle increased 1.75 times at 1 day after injury (*P* < 0.05). PD-1 mRNA peaked at 3 days after injury, which was 4.03 times that of the noninjury group (*P* < 0.05). After that, the expression of PD-1 mRNA began to decline, and at 7 days after injury, the expression of PD-1 mRNA was 1.79 times that of the noninjury group (*P* < 0.05). PD-1 mRNA levels returned to normal at 14 days after the injury (Fig. 1).

### PD-1 knockout impairs contused skeletal muscle regeneration

**Fig. 2:**
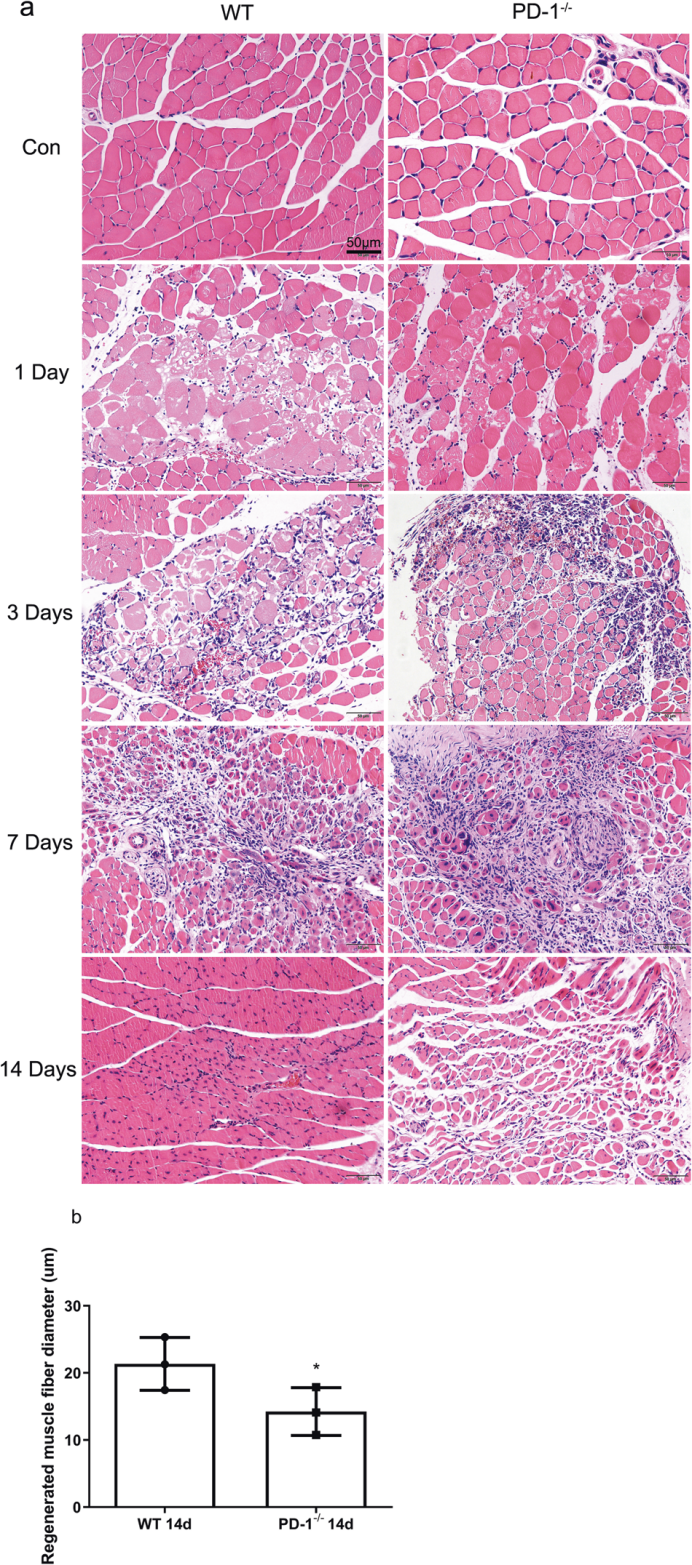
Morphological manifestations of skeletal muscle contusion in wild-type mice and PD-1^−/−^ mice. (**a**) HE staining results. (**b**) Comparison of regenerated muscle fiber diameter at 14 days after injury. Data are mean ± SD, *n* (WT) = 3, *n* (PD-1^−/−^) = 3. *Compared with WT14, *P* < 0.05.

HE staining results showed that the muscle fibers of the WT_con_ and P_con_ groups had regular morphology (Fig. 2a). The nucleus was located under the sarcolemma in each muscle cell, the muscle fiber structure was intact, without necrosis or inflammatory cell infiltration. On the first day after skeletal muscle injury, wild-type mice and PD-1 knockout mice showed a large amount of muscle fiber swelling, necrosis, and inflammatory cell infiltration, but there was no significant difference between the two groups. On the third day after skeletal muscle injury, some central nuclear muscle fibers (regenerative muscle fibers) appeared in wild-type mice, but PD-1 knockout mice showed fewer regenerative muscle fibers than in wild-type mice, and there were many swollen muscle cells and inflammatory cells. On the seventh day after skeletal muscle injury, a large number of regenerated muscle fibers were observed in wild-type mice, and these muscle fibers were relatively large. PD-1 knockout mice only had a small number of regenerated muscle fibers, and there were still a large number of incomplete muscle fiber structures. On the 14th day after skeletal muscle injury, there were only a few regenerated muscle fibers in wild-type mice, while a large number of regenerated muscle fibers were observed in PD-1 knockout mice, and the diameter of the regenerated muscle fibers in PD-1 knockout mice was significantly lower than those in wild-type mice (*P* < 0.05) (Fig. 2b), indicating that PD-1 knockout delayed and impaired skeletal muscle regeneration.

### Effects of PD-1 knockout on contused skeletal muscle matrix remodeling

**Fig. 3:**
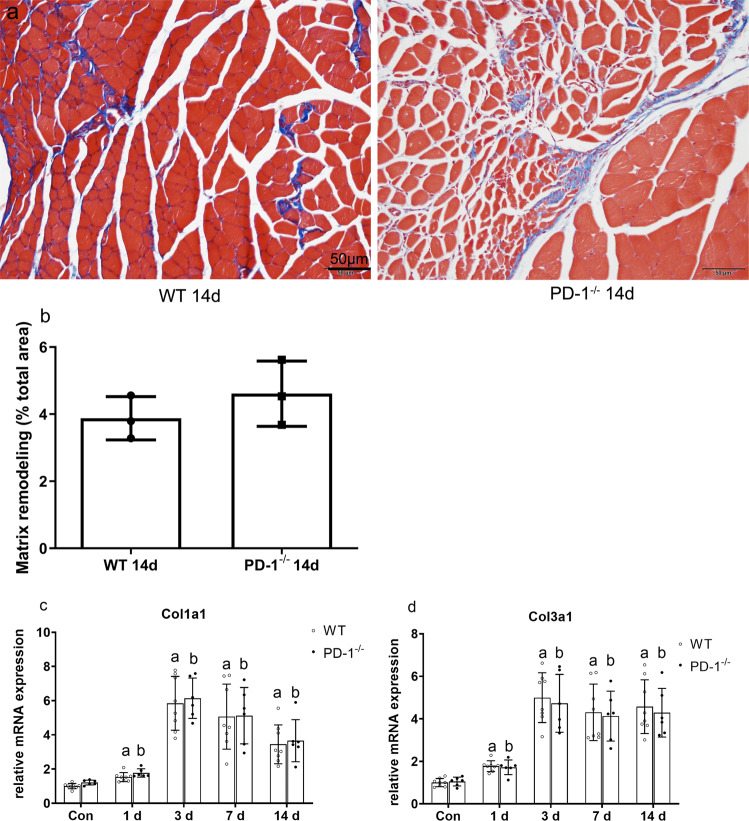
Matrix remodeling in wild-type mice and PD-1^−/−^ mice. (**a**) Masson staining results of wild-type mice and PD-1^−/−^ mice, the red part is myocytoplasm, blue part is matrix remodeling. (**b**) Comparison of blue collagen fiber area to total area at 14 days after injury. (**c**) Col1a1 mRNA expression level at various time points after skeletal muscle contusion. (**d**) Col3a1 mRNA expression level at various time points after skeletal muscle contusion. Data are mean ± SD, *n* (WT) = 8, *n* (PD-1^−/−^) = 6. ^a^compared with WT_con_ group, *P* < 0.05; ^b^compared with P_con_ group, *P* < 0.05.

Masson staining results showed that blue collagen fibers appeared in the skeletal muscles of the WT14 group and P14 group (Fig. 3a), but there was no significant difference in the size of the blue collagen fibers between the WT14 and P14 groups (Fig. 3b, *P* > 0.05).

In addition, the levels of Col1a1 and Col3a1 mRNA in wild-type and PD-1^−/−^ mice were significantly higher than those in the uninjured group at 1, 3, 7, and 14 days after injury (P < 0.05). However, there was no significant difference between the PD-1^−/−^ and wild-type mice (P > 0.05) (Fig. 3c, d). These results show that PD-1 knockout did not affect contused skeletal muscle matrix remodeling.

### Effects of PD-1 knockout on Treg cells in contused skeletal muscle

**Fig. 4:**
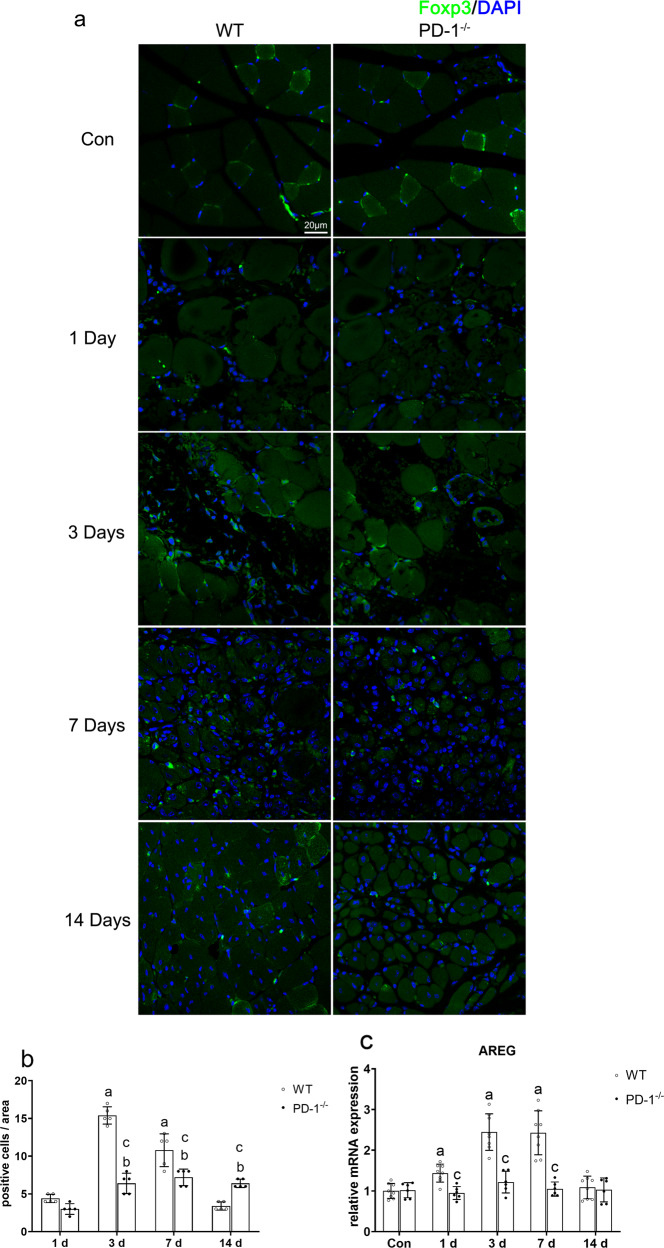
Results of Treg cells in wild-type mice and PD-1^−/−^ group after skeletal muscle contusion. (**a**) Foxp3 immunofluorescence staining of Treg cell markers. (**b**) Average positive cells in a single field of view. (**c**) AREG (specific muscle growth factor secreted by Treg cells) mRNA expression at various time points after skeletal muscle contusion. Data are mean ± SD, *n* (WT) = 8, *n* (PD-1^−/−^) = 6. ^a^Compared with WTcon group or WT1 group, *P* < 0.05; ^b^Compared with Pcon group or P1 group, *P* < 0.05; ^**c**^At the same time point, the PD-1^−/−^ mice compared with wild-type mice, *P* < 0.05.

Immunofluorescence staining for the Treg cell marker Foxp3 [19] (Fig. 4a) showed that at 1 day after skeletal muscle contusion, a small amount of Treg cell infiltration occurred in wild-type mice and PD-1^−/−^ mouse skeletal muscle. There was no significant difference in the number of Treg cells between wild-type and PD-1^−/−^ mice (*P* > 0.05). Three days after skeletal muscle contusion, many Treg cells infiltrated the skeletal muscle of wild-type mice, while the number of Treg cells in PD-1^−/−^ mice increased slightly, and the number of Treg cells in PD-1^−/−^ mice was less than that in wild-type mice (*P* < 0.05). Seven days after skeletal muscle contusion, the number of Treg cells in wild-type mice was reduced, while the number of Treg cells in PD-1^−/−^ mice was not changed, but the number of Treg cells in the PD-1^−/−^ mice was still lower than that in the wild-type group (*P* < 0.05). At 14 days after skeletal muscle contusion, the number of Treg cells in wild-type mice was further reduced and close to normal levels, while the number of Treg cells in the PD-1^−/−^ mice remained unchanged, maintaining a moderate level, but at this time, there were more Treg cells in PD-1^−/−^ mice than in wild-type mice (*P* < 0.05, Fig. 4b).

The AREG mRNA levels in wild-type mice were significantly higher than those in the WT_con_ group at 1, 3, and 7 days after injury (*P* < 0.05). There was no significant difference in the AREG mRNA levels in the PD-1^−/−^ group in comparison to that of the P_con_ group at 1, 3, 7, and 14 days after injury (*P* > 0.05). In addition, AREG mRNA levels in the PD-1^−/−^ mice were significantly lower than those in wild-type mice at 1, 3, and 7 days after injury (*P* < 0.05) (Fig. 4c). These results indicate that PD-1 knockout reduces the number and impairs the function of Treg cells in contused skeletal muscle compared to those of wild-type mice.

### PD-1 knockout increases macrophage pro-inflammatory polarization and decreases anti-inflammatory polarization in contused skeletal muscle

**Fig. 5:**
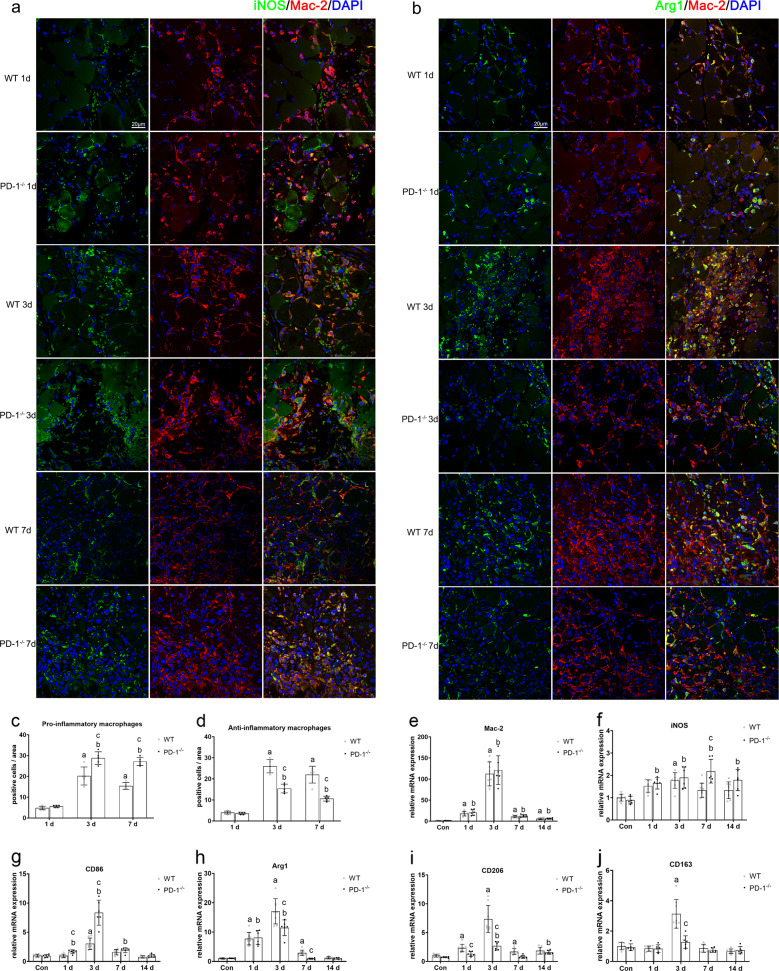
Macrophage polarization in wild-type mice and PD-1^−/−^ mice after skeletal muscle contusion. **a** iNOS immunofluorescence staining of pro-inflammatory macrophage markers. **b** Arg1 immunofluorescence staining of anti-inflammatory macrophage markers. **c** Average pro-inflammatory macrophages in a single field of view. **d** Average anti-inflammatory macrophages in a single field of view. **e** Mac-2 mRNA expression at various time points after skeletal muscle contusion. **f** iNOS mRNA expression at various time points after skeletal muscle contusion. **g** CD86 mRNA expression at various time points after skeletal muscle contusion. **h** Arg1 mRNA expression at various time points after skeletal muscle contusion. **i** CD206 mRNA expression at various time points after skeletal muscle contusion. **j** CD163 mRNA expression at various time points after skeletal muscle contusion. Data are mean ± SD, *n* (WT) = 8, *n* (PD-1^−/−^) = 6. ^a^Compared with WT_con_ group or WT1 group, *P* < 0.05; ^b^Compared with P_con_ group or P1 group, *P* < 0.05; ^c^at the same time point, the PD-1^−/−^ mice compared with wild-type mice, *P* < 0.05.

Immunofluorescence results (Fig. 5a, b) showed that macrophage infiltration in wild-type mice and PD-1^−/−^ mice was apparent 1 day after skeletal muscle contusion; there was no significant difference in the number of pro-inflammatory or anti-inflammatory macrophages between PD-1^−/−^ and WT groups (*P* > 0.05, Fig. 5c, d). Three days after skeletal muscle contusion, the infiltration of wild-type mice and PD-1^−/−^ mouse macrophages further increased, and in the PD-1^−/−^ mice, the number of pro-inflammatory macrophages was greater than that of wild-type mice, while the number of anti-inflammatory macrophages was less than that of wild-type mice (*P* < 0.05). Seven days after skeletal muscle contusion, wild-type mice and the PD-1^−/−^ mice still had significant macrophage infiltration, and the number of pro-inflammatory macrophages in the PD-1^−/−^ mice was greater than that of wild-type mice, while the number of anti-inflammatory macrophages was less than that of wild-type mice (*P* < 0.05).

The mRNA levels of the total macrophage marker Mac-2 [4, 5] in wild-type mice and PD-1^−/−^ mice were significantly higher than those in the noninjured group at 1, 3, 7, and 14 days after injury (*P* < 0 05). However, there was no significant difference between the PD-1^−/−^ and wild-type mice (*P* > 0.05) (Fig. 5e). mRNA levels of the pro-inflammatory macrophage markers, [14, 15] iNOS and CD86, in wild-type mice were significantly higher than those in the WT_con_ group (*P* < 0.05) at 1, 3, and 7 days after injury, expression of these markers was significantly higher than that in the P_con_ group (*P* < 0.05). In addition, iNOS and CD86 mRNA levels in PD-1^−/−^ mice were significantly higher than those in wild-type mice at 3 and 7 days after injury (*P* < 0.05) (Fig. 5f, g). The mRNA levels of the wild-type mouse anti-inflammatory macrophage markers, [14, 15] Arg1, CD206, and CD163, were significantly higher than those in the WT_con_ group at 1, 3, and 7 days after injury (*P* < 0.05). The mRNA levels of Arg1 and CD206 in PD-1^−/−^ mice were significantly higher than those in the P_con_ group at 3 days after injury (*P* < 0.05), and there was no significant difference in CD163 (*P* > 0.05). In addition, Arg1, CD206, and CD163 mRNA levels in the PD-1^−/−^ mice were significantly lower than those in wild-type mice at 3 days after injury (*P* < 0.05) (Fig. 5h–j). These results indicate that PD-1 knockout increased pro-inflammatory polarization and decreased anti-inflammatory polarization in contused skeletal muscle macrophages compared to that of wild-type mice.

### PD-1 knockout promotes contused skeletal muscle inflammation

**Fig. 6:**
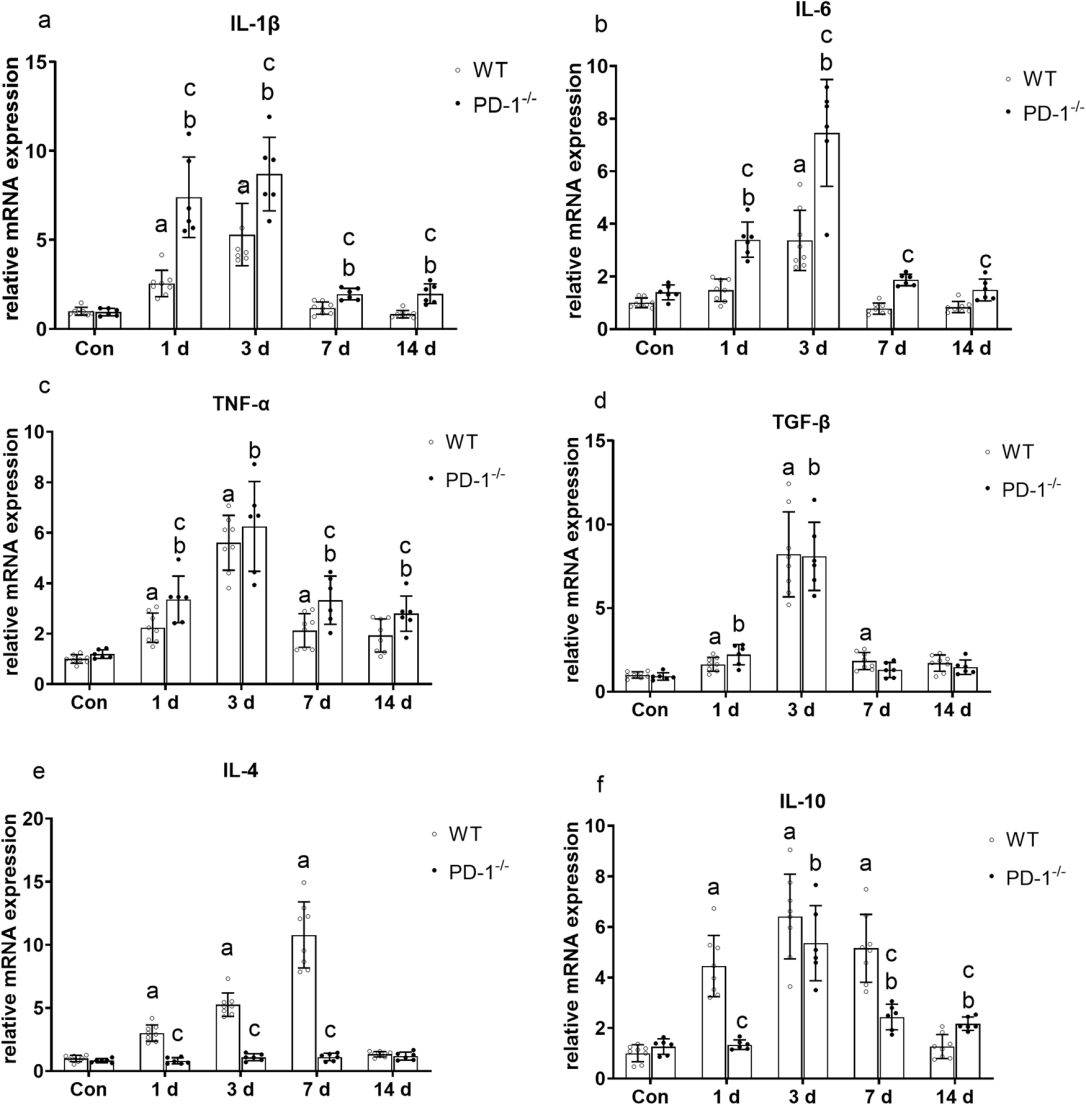
Expression of pro-inflammatory and anti-inflammatory factors in wild-type mice and PD-1^−/−^ mice after skeletal muscle contusion. (**a**) IL-1β mRNA expression at various time points after skeletal muscle contusion. (**b**) IL-6 mRNA expression at various time points after skeletal muscle contusion (**c**) TNF-α mRNA expression at various time points after skeletal muscle contusion. (**d**) Skeletal muscle TGF-β mRNA expression at various time points after skeletal muscle contusion (**e**) IL-4 mRNA expression at various time points after skeletal muscle contusion. (**f**) IL-10 mRNA expression at various time points after skeletal muscle contusion. Data are mean ± SD, *n* (WT) = 8, *n* (PD-1^−/−^) = 6. ^a^Compared with WT_con_ group, *P* < 0.05; ^b^Compared with P_con_ group, *P* < 0.05; ^c^at the same time point, the PD-1^−/−^ mice compared with wild-type mice, *P* < 0.05.

The mRNA levels of IL-1β, IL-6, and TNF-α in wild-type mice were significantly higher than those in the WT_con_ group at 3 days after injury (*P* < 0.05). The mRNA levels of IL-1β, IL-6, and TNF-α in PD-1^−/−^ mice were significantly higher than those in the P_con_ group at 1 and 3 days after injury (*P* < 0.05). In addition, the mRNA levels of IL-1β, IL-6, and TNF-α in PD-1^−/−^ mice were significantly higher than those in wild-type mice at 1, 7, and 14 days after injury (*P* < 0.05) (Fig. 6a–c). The mRNA levels of the anti-inflammatory factors IL-4, TGF-β, and IL-10 in wild-type mice were significantly higher than those in the WT_con_ group at 1, 3, and 7 days after injury (*P* < 0.05). The mRNA levels of TGF-β and IL-10 in PD-1^−/−^ mice were significantly higher than those in the P_con_ group (*P* < 0.05), and there was no significant difference in IL-4 (*P* > 0.05). In addition, the mRNA levels of IL-4 and IL-10 in the PD-1^−/−^ mice at 1 and 7 days after injury were significantly lower than those in wild-type mice (*P* < 0.05), and there was no significant difference in TGF-β (*P* > 0.05). (Fig. 6d–f). These results indicate that PD-1 knockout resulted in increased inflammation in contusion skeletal muscle compared to that of wild-type.

### PD-1 knockout promotes oxidative stress in contused skeletal muscle

**Fig. 7:**
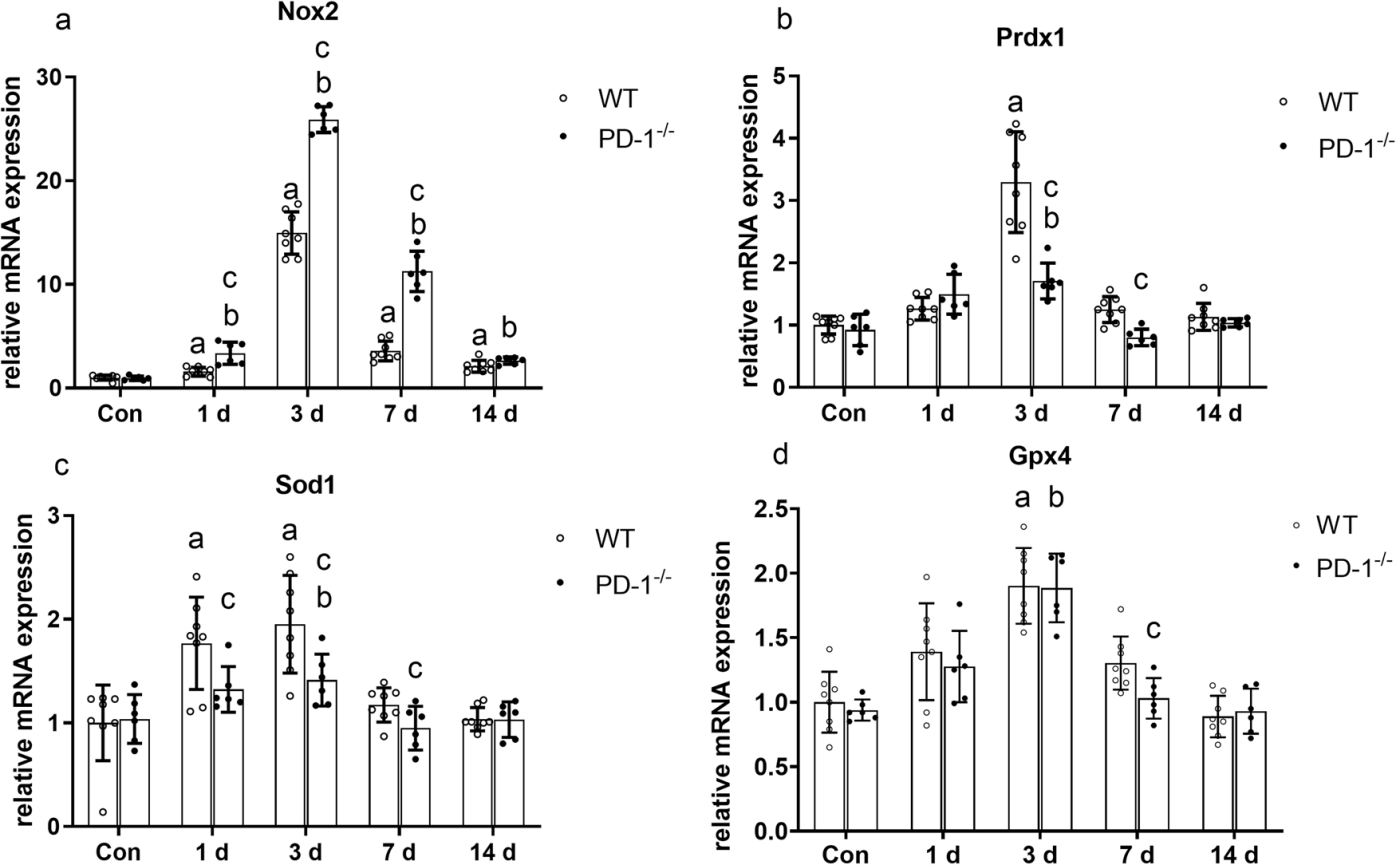
Expression of oxidative stress-related factors in wild-type mice and PD-1^−/−^ mice after skeletal muscle contusion. (**a**) Nox2 mRNA expression at various time points after skeletal muscle contusion. (**b**) Prdx1 mRNA expression at various time points after skeletal muscle contusion. (**c**) sod1 mRNA expression at various time points after skeletal muscle contusion. (**d**) Gpx4 mRNA expression at various time points after skeletal muscle contusion. Data are mean ± SD, *n* (WT) = 8, *n* (PD-1^−/−^) = 6. ^a^Compared with WT_con_ group, *P* < 0.05; ^b^Compared with P_con_ group, *P* < 0.05; ^c^at the same time point, the PD-1^−/−^ mice compared with wild-type mice, *P* < 0.05.

The mRNA levels of the oxidative stress factor Nox2 in wild-type and PD-1^−/−^ mice were significantly higher than those in the noninjury group at 1, 3, 7, and 14 days after injury (*P* < 0.05). In addition, Nox2 mRNA levels were significantly higher in PD-1^−/−^ mice than in wild-type mice at 1, 3, and 7 days after injury (*P* < 0.05) (Fig. 7a). The mRNA levels of the antioxidative stress factors Prdx1, sod1, and Gpx4 in wild-type and PD-1^−/−^ mice were significantly higher than those in the noninjury group at 3 days after injury (*P* < 0.05). In addition, the mRNA levels of Prdx1, sod1, and Gpx4 in PD-1^−/−^ mice at 3 and 7 days after injury were significantly lower than those of wild-type mice (*P* < 0.05) (Fig. 7b–d). These results indicate that PD-1 knockout increased oxidative stress in contused skeletal muscle compared to that of wild-type.

### PD-1 knockout reduces regenerative factors in contused skeletal muscle

**Fig. 8:**
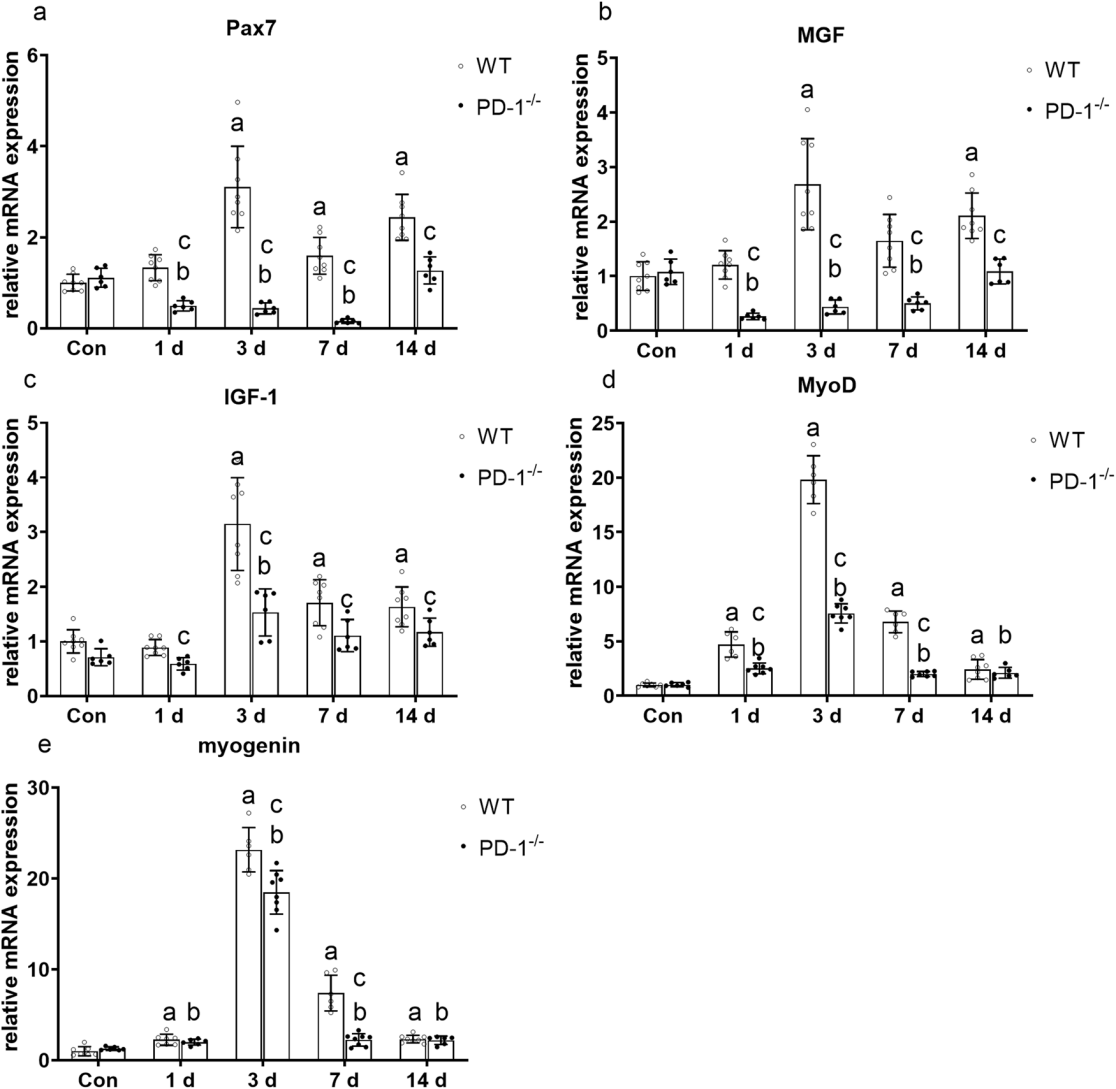
Expression of muscle regeneration factors in wild-type mice and PD-1^−/−^ mice after skeletal muscle contusion. (**a**) Pax7 mRNA expression at various time points after skeletal muscle contusion. (**b**) MGF mRNA expression at various time points after skeletal muscle contusion. (**c**) IGF-1 mRNA expression at various time points after skeletal muscle contusion. (**d**) MyoD mRNA expression at various time points after skeletal muscle contusion. (**e**) Myogenin mRNA expression at various time points after skeletal muscle contusion. Data are mean ± SD, *n* (WT) = 8, *n* (PD-1^−/−^) = 6. ^a^Compared with WTcon group, *P* < 0.05; ^b^Compared with Pcon group, *P* < 0.05; ^c^at the same time point, the PD-1^−/−^ mice compared with wild-type mice, *P* < 0.05.

The mRNA levels of Pax7, MGF, IGF-1, MyoD, and myogenin in wild-type mice were significantly higher than those in the WT_con_ group at 3 and 14 days after injury (*P* < 0.05). At 1, 3, and, 7 days after injury, expression of Pax7 and MGF were significantly lower than that of the P_con_ group (*P* < 0.05), and IGF-1, MyoD, and myogenin were increased at 1, 3, 7, or 14 days after injury compared to that of the P_con_ group (*P* < 0.05). In addition, Pax7, MGF, IGF-1, MyoD, and myogenin mRNA levels in PD-1^−/−^ mice were significantly lower than those in wild-type mice at 1, 3, 7, or 14 days after injury (*P* < 0.05) (Fig. 8a–e). These results indicate that PD-1 knockdown inhibits regenerative muscle factors in contused skeletal muscle compared to that of wild-type contused skeletal muscle.

## Discussion

To study the role of PD-1 in Treg cells in skeletal muscle regeneration, we established a skeletal muscle contusion model and a PD-1 knockout mouse model. The morphological results showed that wild-type mice had recovered to nearly normal levels on day 14 after skeletal muscle injury, but this was not the case in the PD-1^−/−^ mice. In PD-1 knockout mice, the number of infiltrating inflammatory cells in injured skeletal muscles increased, the duration was prolonged, the number of regenerated muscle fibers was reduced, the generation time was delayed, and the diameter of regenerated muscle fibers was smaller than wild-type mice at 14 days after injury. These results suggest that PD-1 knockout impairs regeneration of contused skeletal muscle. In addition, we studied the effects of PD-1 knockout on injured skeletal muscle matrix remodeling. Masson staining and expression of Col1a1, Col3a1, and TGF-β showed that fibrotic repair occurred during the repair of contused skeletal muscle. This is consistent with the results of previous studies [36, 37], but PD-1 knockout did not affect skeletal muscle matrix remodeling, and the specific reason needs further study and explanation.

Treg cells are a type of CD4^+^ CD25^+^ T lymphocyte that specifically express Foxp3 [19] and participate in various immune and inflammatory responses in the body [21, 22]. Treg cells also express PD-1, which promotes the production of pTreg cells in peripheral tissues and plays an important role in maintaining the normal function of pTreg cells [21, 28–30]. This study found that the number of Treg cells increased in the early stage (1–3 days) of skeletal muscle injury in wild-type mice and decreased to normal levels in the late stage (7–14 days). This finding is consistent with previous reports. For example, Burzyn reported that the number of Treg cells increased after skeletal muscle injury, which is helpful for the repair of injured skeletal muscle [20]. The number of Treg cells in injured skeletal muscle in PD-1 knockout mice was reduced compared to that of wild-type mice, and the number of Treg cells did not return to normal levels at 14 days after injury. This shows that PD-1 knockout reduces the generation of Treg cells and impairs the regeneration of contused skeletal muscle. These results are similar to those of another study, which found that after inhibiting the PD-1 pathway, Treg cell production and development in peripheral tissues was inhibited, indicating that PD-1 is essential for the production and development of peripheral tissues [21]. In addition, compared with the uninjured group, the number of Treg cells increased after PD-1^−/−^ mice skeletal muscle injury, but there was no significant expression change in specific muscle growth factor AREG [19, 20, 23, 25] secreted by Treg cells, indicating that PD-1 knockout may also affect the function of Treg cells. Therefore, these results suggest that after PD-1 knockout, the number of Treg cells in injured skeletal muscle is reduced, and the function is impaired, which may impair contused skeletal muscle regeneration.

Macrophages play an important role in skeletal muscle regeneration. It is still unknown whether they are involved in PD-1 knockout-induced impairment of contused skeletal muscle regeneration. We studied this issue. In the present study, Mac-2 was used as a marker of total macrophages [4, 5], iNOS and CD86 were used as pro-inflammatory macrophage markers [14, 15], and Arg1, CD206, and CD163 were used as anti-inflammatory macrophage markers [14, 15]. The present study found that the number of total macrophages, pro-inflammatory macrophages, and anti-inflammatory macrophages increased in the early stage (1–3 days) of skeletal muscle injury in wild-type mice. In the later stages (7–14 days) of skeletal muscle injury, all cell types were reduced. After PD-1 knockout, the number of pro-inflammatory macrophages was significantly increased compared with that of wild-type mice (while the pro-inflammatory-associated inflammatory factors IL-1β, IL-6, TNF-α were enhanced), while the number of anti-inflammatory macrophages decreased significantly. PD-1 knockout led to increased pro-inflammatory and decreased anti-inflammatory macrophage polarization, thereby impairing skeletal muscle regeneration. This result is similar to many studies that showed that after PD-1 knockout in mice, peritoneal macrophages [15] and macrophages/microglia [14] increased pro-inflammatory polarization and decreased anti-inflammatory polarization.

We further explored the mechanism by which PD-1 knockout increased pro-inflammatory polarization and decreased anti-inflammatory polarization. These results showed that as compared to wild-type mice, PD-1 knockout mice IL-4, IL-10 expression was significantly reduced. Furthermore, macrophages increased pro-inflammatory polarization and anti-inflammatory polarization. Previous studies have shown that IL-4 and IL-10 are important factors for inducing anti-inflammatory polarization of macrophages [14, 38, 39]. These findings suggest that PD-1 knockout reduces the expression of IL-4 and IL-10, which may block the macrophage pro-inflammatory-to-anti-inflammatory switch and damage the regeneration of contused skeletal muscle.

Furthermore, we explored the role of oxidative stress factors in PD-1 knockout-induced impairment of contused skeletal muscle regeneration. In the early stage of skeletal muscle injury, the upregulation of oxidative stress factors is conducive to the elimination of necrotic muscle fibers; in the later stage of skeletal muscle injury, the upregulation of antioxidative stress factors is beneficial in reducing oxidative stress and promoting injured skeletal muscle regeneration [40]. This is in line with our previous research results. After skeletal muscle contusion, higher oxidative stress levels are significantly associated with impaired skeletal muscle regeneration [36, 37]. In the present study, the expression of oxidative stress factor (Nox2) and antioxidative stress factors (Prdx1, sod1, and Gpx4) in the early stage of skeletal muscle injury in wild-type mice was significantly upregulated, and in the later period, it was downregulated to close to normal levels. The expression of oxidative stress factors in PD-1 knockout mice increased compared to that of wild-type mice, while the expression of antioxidative stress factors decreased significantly; that is, an oxidative stress imbalance occurred, which may impair contused skeletal muscle regeneration.

In addition, muscle regeneration-related factors also play an important role in the regeneration process in contused skeletal muscle, and we conducted related research on this process. The results showed that expression of a variety of muscle regeneration factors (Pax7, MGF, IGF-1, MyoD, and myogenin) was significantly upregulated after skeletal muscle injury in wild-type mice and downregulated to near normal levels in the late regeneration period. After PD-1 knockout, the expression of these muscle regeneration-related factors was significantly upregulated and was significantly lower than that in wild-type mice, and some factors (Pax7 and MGF) were even lower than those in the uninjured control group. While Pax7 is a marker of muscle satellite cells, MGF and IGF-1 have multiple effects of promoting myoblast proliferation and protein synthesis [41–43]. In addition, MyoD is a marker of proliferation in satellite cells, and myogenin is a marker of differentiation in satellite cells [36]. These factors play an important role in promoting the regeneration of contused skeletal muscle, and their reduced expression may affect skeletal muscle regeneration. In addition, studies have shown that macrophages infiltrated in injured skeletal muscle can express large amounts of IGF-1, which is an important source of IGF-1. These macrophage-derived IGF-1 is considered as a key factor in inflammation resolution and macrophage polarization during muscle regeneration [44]. This is also in line with the results of this article. Compared with wild-type mice, PD-1 knockout mice have significantly lower IGF-1 expression, reduced M2 macrophage polarization, and increased inflammation levels. Therefore, oxidative stress factors and muscle regeneration factors play an important role in the process by which PD-1 knockout impairs contused skeletal muscle regeneration.

In summary, PD-1 knockout reduced the number of Treg cells and blocked the macrophage pro-inflammatory-to-anti-inflammatory switching in contused skeletal muscle, as manifested by the downregulation of muscle regeneration factors, prolonged inflammatory response period, exacerbated oxidative stress and impaired contused skeletal muscle regeneration. These results indicate that PD-1 can promote contused skeletal muscle regeneration by regulating Treg cell generation and macrophage polarization.

## Data Availability

The data that support the findings of this study are available from the corresponding author upon reasonable request.
